# A State of the Art of Antioxidant Properties of Curcuminoids in Neurodegenerative Diseases

**DOI:** 10.3390/ijms22063168

**Published:** 2021-03-20

**Authors:** Serena Silvestro, Cinzia Sindona, Placido Bramanti, Emanuela Mazzon

**Affiliations:** IRCCS Centro Neurolesi “Bonino-Pulejo”, Via Provinciale Palermo, Contrada Casazza, 98124 Messina, Italy; serena.silvestro@irccsme.it (S.S.); cinzia.sindona@irccsme.it (C.S.); placido.bramanti@irccsme.it (P.B.)

**Keywords:** neurodegenerative diseases, curcuminoids, anti-oxidant properties

## Abstract

Neurodegenerative diseases represent a set of pathologies characterized by an irreversible and progressive, and a loss of neuronal cells in specific areas of the brain. Oxidative phosphorylation is a source of energy production by which many cells, such as the neuronal cells, meet their energy needs. Dysregulations of oxidative phosphorylation induce oxidative stress, which plays a key role in the onset of neurodegenerative diseases such as Alzheimer’s disease (AD), Parkinson’s disease (PD), and amyotrophic lateral sclerosis (ALS). To date, for most neurodegenerative diseases, there are no resolute treatments, but only interventions capable of alleviating the symptoms or slowing the course of the disease. Therefore, effective neuroprotection strategies are needed. In recent years, natural products, such as curcuminoids, have been intensively explored and studied for their therapeutic potentials in several neurodegenerative diseases. Curcuminoids are, nutraceutical compouns, that owen several therapeutic properties such as anti-oxidant, anti-inflammatory and neuroprotective effects. In this context, the aim of this review was to provide an overview of preclinical and clinical evidence aimed to illustrate the antioxidant effects of curcuminoids in neurodegenerative diseases. Promising results from preclinical studies encourage the use of curcuminoids for neurodegeneration prevention and treatment.

## 1. Introduction

Neurological diseases affect millions of people around the world [1]. The environmental alterations in the central nervous system (CNS) induce the activation of microglia and astrocytes, cells involved in maintaining homeostasis of the CNS. Following damage, these cells activate a response that induces the release of pro-inflammatory cytokines responsible for a local inflammatory reaction [2]. Furthermore, once active, microglia and astrocytes can produce reactive oxygen species (ROS). However, the brain, despite accounting for only 2% of total body weight, consumes about 20% of the body’s oxygen (O_2_); thus, it is rich in the antioxidants involved in preventing the formation of ROS. Excessive ROS production, if not effectively counteracted by cellular constituents, causes pathological conditions such as neurodegenerative diseases [3,4,5]. This is particularly relevant in neurodegenerative diseases such as Alzheimer’s disease (AD), Parkinson’s disease (PD) and amyotrophic lateral sclerosis (ALS). Indeed neuroinflammation and oxidative stress are common features of neurodegenerative diseases [6,7]. Although therapies that delay or control symptoms are available for these disorders, there are currently no effective treatments available. Therefore, there is a growing interest in natural compounds that possess antioxidant and anti-inflammatory properties [8,9].

Curcuma is a spice extracted from the rhizomes of *Curcuma Longa*, belonging to the Zingiberaceae family, cultivated in India and in southeast Asia [10]. The main constituent of Curcuma is curcumin, a yellow pigment commonly used as a spice and food coloring. It is used as a natural medicine for the treatment of inflammatory diseases [11]. Indeed, curcumin is a nutriceutical compound that possesses numerous therapeutic properties against various diseases, including neurodegenerative diseases [12].

Among the several properties of curcuminoids [13,14,15], the anti-inflammatory [16,17] and antioxidant activities [18,19] are the most investigated due to their role in the pathogenesis of neurodegenerative diseases [20]. Indeed, curcuminoids play a key role in the inhibition of enzymes such as p38 Mitogen-activated protein kinase (MAPK) and c-Jun N-terminal kinase (c-JNK) and transcription factors (such as nuclear factor-κB, NF-κB) related to inflammatory processes. Also, they decrease the expression of several pro-inflammatory cytokines [21,22]. Moreover, many of curcumin’s beneficial effects can be associated with its antioxidant properties [23]. Since oxidative stress, caused by excessive production of reactive oxygen species, lipid peroxidation and oxidative damage to DNA and proteins, is responsible for many pathological complications such as neurodegenerative diseases, curcumin could play an important role in these conditions.

Curcumin has been extensively investigated due to its beneficial properties against several neurological diseases such as PD [24], AD [25] and ALS [26].

The purpose of this review is to describe the antioxidant properties of curcuminoids associated with the efficacy in neurological disease. In the present review, preclinical and clinical evidence highlighting the curcuminoids’ antioxidants effects in neurological disorders are summarized.

## 2. Curcumin

**Figure 1:**
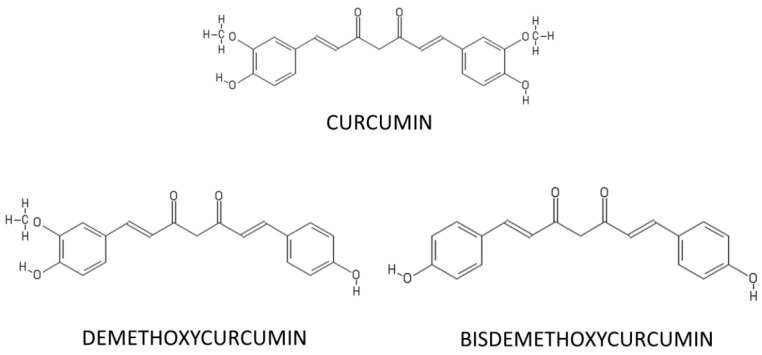
Chemical structure of curcumin and other curcuminoids such as Demethoxycurcumin and Bisdemethoxycurcumin, obtained from the rhizomes of *Curcuma Longa*.

Curcuminoids are the active compounds found in turmeric and include curcumin (diferuloyl methane), demethoxycurcumin and bisdemethoxycurcumin (Figure 1). Several in vitro and in vivo evidence describes the anti-inflammatory [27] and antioxidant properties [28] of curcuminoids. Among the curcuminoids, curcumin makes up about 90% of the curcuminoids present in turmeric. Curcumin is a polyphenol, with the molecular formula C_21_H_20_O_6_, characterized for the first time in 1910 [29].

Among them, curcumin is known to have neuroprotective effects and its antioxidant and anti-inflammatory power is widely studied. The diketone group and two phenolic rings in its chemical structure act as electron traps, thus preventing the production of hydrogen peroxide (H_2_O_2_), hydroxyl radical (OH·) and superoxide anion (O^2−^·). Moreover, the b-diketone fraction and hydroxyl groups of curcumin are capable of complexing metals, such as copper (Cu^2+^) and zinc (Zn^2+^), ferrous iron (Fe^2+^) [30]. Fe^2+^ is necessary for the Fenton reaction that generates OH· radicals; thus, curcumin by chelating the Fe^2+^ protects against metal-induced toxicity. Indeed, it is well known that curcumin contains antioxidant properties and it appears ten times more powerful than vitamin E as a free radical scavenger [31]. Due to the ability of curcumin to cross the blood–brain barrier [32], it also decreases the ROS level [33], protects the brain from lipid peroxidation and reduces the neuron death induced by oxidative insults [34]. Furthermore, curcumin, in addition to acting as direct scavenging of ROS, exerts its antioxidant properties by increasing glutathione (GSH) levels [35], improving the activity of glutathione peroxidase (GSH-Px), glutathione reductase (GR), catalase (CAT) and superoxide dismutase (SOD) [36]. Furthermore, curcumin reduces mitochondrial damage and apoptosis mediated by lipid peroxidation [37].

Curcumin can inhibit the activation of several transcription factors including NF-κB [38], activated protein-1 (AP-1) [39], Notch-1 [40], β -catenin [41] and peroxisome proliferator-activated receptor-gamma (PPAR-γ) [42], all involved in different biological processes, including inflammation. NF-κB is localized in the cytosol—after activation, it is translocated to the nucleus, where it promotes the expression of different genes involved in cell proliferation and inflammation [43]. The dysregulated activity of NF-κB underlies several inflammatory diseases, such as neurodegenerative diseases [44]. Curcumin inhibits NF-κB activation by suppressing the translocation of p65 and the inhibitor κB (IκB) into the nucleus [45]. In this way, it inhibits the activation of numerous genes involved in cell survival, including Bcl-2, Bcl-xL, cyclin D1, cyclooxygenase 2 (COX-2) and matrix metallopeptidase (MMP)-9, thus promoting the arrest cell cycle, inhibiting proliferation and inducing apoptosis [46].

Many of curcumin’s effects are also mediated by its ability to inhibit several protein kinases (PKs) involved in various cellular processes, such as autophosphorylation-activated (AK) protein kinase [47], Ca^2+^-dependent protein kinase (CDPK) [48], Janus kinase (JAK) [49], mitogen-activated protein kinase (MAPK) [50,51], the mammalian target of rapamycin (mTOR) [52,53], phosphorylase kinase (PhK) [47], cytosolic protein kinase (cPK) [47], PKA [47], PKB/Akt [54], PKC [47]. Furthermore, the inhibitory action on the MAPK pathway and the inhibition of the phosphorylation of the extracellular receptor kinase (ERK), c-JNKs and p38 MAPKs are responsible for the powerful anti-inflammatory effects of curcumin [55]. Indeed, the inhibition of the NF-κB and MAPK pathways reduce the expression of numerous inflammatory interleukins (ILs) such as IL-1β, IL-6, Tumor necrosis factor-alpha (TNF-α) [56], IL-2 [57], IL-5 [58], IL-8 [59], IL-12 [60], IL-18 [61] and signal transducer and activator of transcription (STAT) proteins [49]. Therefore, curcumin by regulating transcription factors, such as NF-κB, inhibits the production of pro-inflammatory cytokines, thus exhibiting a powerful anti-inflammatory action [62].

These characteristic properties of curcumin make it a valid neuroprotective compound. Indeed, epidemiological studies show that in the Indian population, the consumption of turmeric is closely correlated with the low incidence of neurodegenerative diseases such as AD and PD, compared to the Caucasian population [63,64].

## 3. Methodology

**Figure 2:**
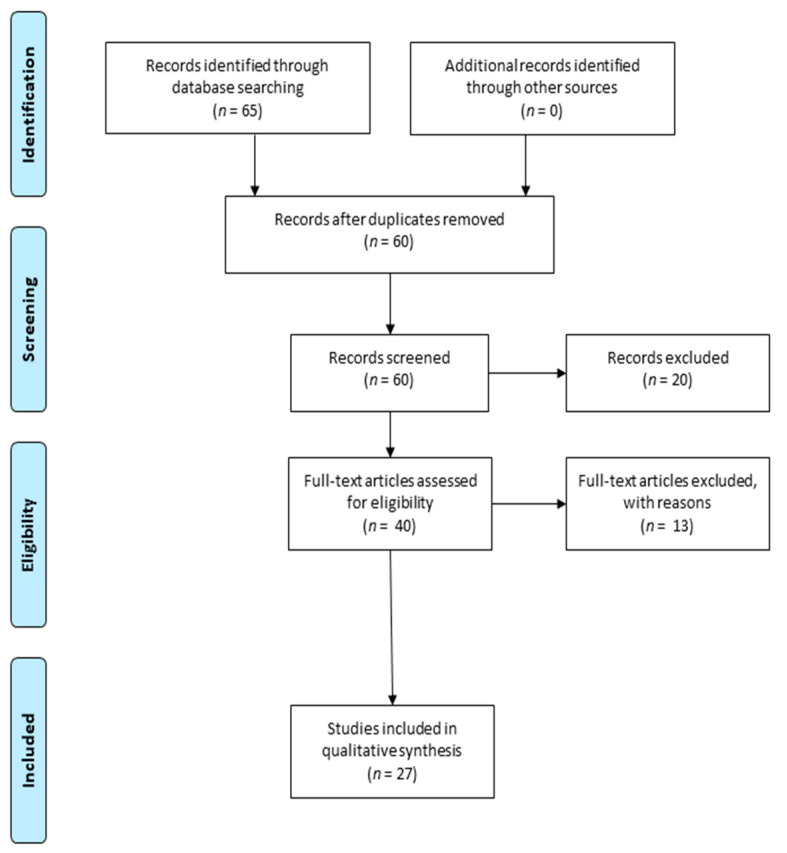
Prisma flow diagram illustrating the selection methodology of the preclinical studies used for the writing of the review. Duplicate articles were excluded from the total of the studies recorded. Instead, they were considered articles that evaluate the antioxidant effects of curcuminoid in neurological disease. The PRISMA Statement was published in [65].

The aim of this manuscript was to provide an overview of experimental and clinical studies that report the antioxidant effects of curcuminoids in neurodegenerative diseases, such as PD, AD and ALS. In order to write this review, a search was carried out in PubMed using the following keywords: “Curcuminoids”, “antioxidant effects”, “Parkinson’s Disease”, “Alzheimer’s Disease”, “Amyotrophic Lateral Sclerosis”. We considered articles published between 2015 and 2021 demonstrating a neuroprotective role of curcuminoids. In this search, 65 articles were found, as shown in the Prisma flow diagram (Figure 2). In the “Records screened” section, 20 articles were excluded; among them, 12 were not considered because they were different from the focus of our review. Two other articles were excluded because they focused on the antioxidant role of other compounds. Moreover, since we focused on the curcuminoids, we also excluded six articles that evaluated the effects of curcumin combined with other compounds. As this review is intended to provide an overview of experimental studies, in the “Full-text articles assessed for eligibility” section, 13 articles were excluded because they are reviews. Finally, in this manuscript, were considered 27 studies that evaluate the biochemical and molecular mechanisms underlying the antioxidant effects of curcuminoids and their therapeutic application in neurological diseases.

In order to support the findings of pre-clinical studies, we also provide a summary of current clinical trials recorded on ClinicalTrial.gov (https://clinicaltrials.gov/ accessed on 12 February 2021), using the following keywords: “Curcumin”, “Parkinson’s Disease” or “Alzheimer’s Disease” or “Amyotrophic Lateral Sclerosis”.

## 4. Parkinson’s Disease

PD is a progressive and neurodegenerative disease induced by the progressive loss of neurons in the substantia nigra, followed by a decrease in dopamine levels in the striatum [66]. These events lead to a dysfunction of the nigrostriatal pathway which consequent movement disorders [67]. The oxidative stress and inflammation response play a key role in contributing to or exacerbating the nigrostriatal degeneration [68]. The distinctive clinical features of PD are tremor, bradykinesia, rigidity, and balance disturbances. Additionally, in patients with PD also occurs non-motor deficits such as anxiety, dementia, depression, sleep disturbances, and psychosis that negatively condition the quality of life [69]. Moreover, the hallmark of PD is the formation of Lewy bodies, which are cytoplasmic inclusions of alpha-synuclein (α-syn) [70]. However, oxidative stress and inflammation play a key role in PD. In 90% of cases, PD occurs in a sporadic form; only 10% of cases present as a familial form. Familial forms of PD involve α-synuclein gene mutations (*SNCA*), ubiquitin C-terminal hydrolase L1 (*UCHL-1*), phosphatase and tensin homolog-induced putative kinase 1 (*PINK1*), Parkin (*PRKN*), protein deglycase (*DJ-1*), and leucine-rich repeat kinase 2 (*LRRK2*) [71].

To date, treatments for PD include drugs such as 1,3,4-dihydroxyphenylalanine (L-DOPA) or anticholinergic, which help relieve symptoms, but are not effective. In addition, many of these drugs have side effects that limit their use [72]. Therefore, effective and safe therapeutic interventions are necessary. In this context, interest is growing in the use of curcuminoids, which own antioxidant and anti-inflammatory properties, as alternative therapies.

### 4.1. Anti-Oxidant Effects of Curcuminoids In Vitro Models of PD

Several in vitro evidence has shown that curcumin has antiparkinsonian effects due to its antioxidant [73], anti-inflammatory [74], and anti-apoptotic properties [75], as well as its protective effects against mitochondrial damage [76].

Yu et al. evaluated the effects of curcumin through an in vitro experiment in primary mesencephalic astrocytes treated with 1-methyl-4-phenylpyridinium ion (MPP^+^) to induce a PD model. The cells were pre-treated with increasing curcumin concentrations (0–16 μM) for 48 h. Through the vitality assay, concentrations greater than 8 μM were found to induce a reduction in cell viability; therefore, 8 μM was chosen as the suitable concentration. Immunofluorescence analysis revealed that pre-treatment with curcumin did not cause significant morphological changes in the astrocytes but reduced their MPP^+^-induced activation. Furthermore, oxidative stress conditions were assessed through the measurement of ROS and GSH levels in astrocytes, which are determinants in parkinsonian conditions. Curcumin substantially caused a reduction of ROS and an increase of GSH, compared to the primary astrocytes treated with MPP^+^.

The anti-inflammatory property of curcumin was also evaluated in the present study, focusing in particular on Toll-like receptor 4 (TLR4), which appears to play a key role in PD neurophysiopathology. Pre-treatment with curcumin reduced the MPP^+^ induced toxicity by reducing the expression of MyD88 and TRIF, inhibiting the TLR4 pathway, a common MyD88-dependent signaling pathway. Curcumin significantly inhibited the NF-κB and IRF3 activation and reduced the TLR4 levels. In this way, the curcumin exerted its anti-inflammatory effects through inhibition of TLR4 and its downstream signaling pathway.

In conclusion, the findings of this experiment highlighted both the antioxidants and the anti-inflammatory properties exerted from curcumin, in MPP^+^-treated mesencephalic astrocytes [77]. Uğuz A.C. et al. studied the molecular effects of curcumin on several intracellular signaling pathways in the cellular model of oxidative stress. Specifically, the researchers evaluated the effect of curcumin on Ca^2+^ signaling, measurement of ROS, mitochondrial depolarization levels and caspase-3 and -9 activities in SH-SY5Y neuronal cells treated with H_2_O_2_. Curcumin protected the cells from H_2_O_2_-induced apoptosis. It reduced lipid peroxidation and intracellular Ca^2+^ concentrations compared to the H_2_O_2_-treated group of cells. Conversely, curcumin treatment increased GSH and GSH-Px levels. Furthermore, the increased H_2_O_2_-induced caspase-3 and caspase-9 expression were reduced by curcumin, thus protecting neuronal cells from oxidative damage [78]. Dehghani Z. et al. showed that curcumin (25 and 50 μM), in a dose-dependent manner, reduced the growth of α-syn fibrils in rat brain mitochondria. It has been observed that curcumin reduced the cytotoxicity of α-syn aggregates by reducing the release of mitochondrial Type 1 Hexokinase and ROS formation induced by α-syn fibrillation products [79].

Instead, Ramkumar et al. evaluated the demethoxycurcumin (DMC), a derivative of curcumin in an in vitro model of PD induced by rotenone (ROT), a phytoactive compound that causes toxicity in neuronal cells. Two hours prior to ROT (100 nM) treatment, SH-SY5Y neuroblastoma cells were pre-treated with DMC (5 nM, 10 nM, 20 nM, 50 nM, 100 nM, 200 nM, 500 nM, and 1 μM). The treatment showed that DMC doses higher than 50 nM were toxic to SH-SY5Y cells; thus, the dose of 50 nM of DMC was chosen for further investigation. The results showed that DMC increased cell viability and its induced a decrease in both MMP and apoptotic process. In this regard, immunoblotting analysis was performed to evaluate the expression of apoptotic proteins in the mitochondria and in the cytosol, detecting that, DMC increased the Bcl-2, Bcl-xL level in the mitochondria. Moreover, the analysis found that pre-treatment with DMC restored the expression of increased pro-apoptotic proteins following exposure to ROT. DMC improved the ROT-induced oxidative stress through a significantly reduced ROS level in SH-SY5Y cells. In conclusion, these data highlighted that pre-treatment with DMC inhibited the apoptosis process reducing pro-apoptotic proteins, increasing anti-apoptotic proteins, and ameliorating oxidative stress [80].

The in vitro study conducted by Buratta et al. proves the therapeutic and antioxidant properties of curcumin. PC12 cells were treated simultaneously with ROT (0.1–1 μM) and curcumin (10 μM) for 24 h. PC12 treated curcumin increased cell viability and reduced oxidative stress induced by ROT treatment. Furthermore, the authors investigated the effect of curcumin in carbonylation and nitrosation of proteins and the activity of the proteasome. Immunoblot investigations showed that curcumin significantly reduced carbonylation and protein tyrosine nitration. PC12 treated with ROT reduced the activity of the proteasome, which is reflected in a concomitant increase in oxidized proteins. Conversely, treatment with curcumin in PC12 counteracted inhibition of proteasomal activity ROT-induced, restoring the oxidized protein levels. The prevent proteasome activity could be another beneficial effect used from curcumin for exerting its antioxidant properties [81].

### 4.2. Anti-Oxidant Effects of Curcuminoids in In Vivo Models of PD

In an in vivo study performed on adult male Sprague-Dawley rats injured unilaterally with 6-hydroxydopamine (6-OHDA) in the left striatum to induce the PD model, curcumin increased the levels of the dopamine transporter (DAT) and tyrosine hydroxylase (TH). Contrarily, it reduced the levels of the glial fibrillar acid protein (GFAP). In this way, curcumin could reduce the local tissue damage induced by 6-OHDA. Furthermore, curcumin-treated rats showed a significant increase in Wnt3a and β-catenin mRNA expression. Wnt/β-catenin signaling is involved in neuronal survival, differentiation, axonal extension, promotes neurogenesis, synapse formation, and plasticity, and induces neuroprotection. Therefore, by activating the Wnt/β-catenin signaling pathway curcumin could improve the vitality and survival of neuronal cells. 

Furthermore, it was shown that activation of the Wnt/β-catenin pathway also increased the levels of endogenous antioxidant molecules such as GSH-Px, SOD, and reduced the concentration levels of malondialdehyde (MDA). Therefore, curcumin showed antioxidant effects against 6-OHDA injury in PD rats through activation of the Wnt/β-catenin signaling pathway [82]. 

Despite its beneficial effects, curcumin is a dietary polyphenol with poor bioavailability. In this regard, Hirata et al. evaluated the potential antioxidant effect of GIF-2165X-G1, a hybrid molecule containing an oxindole skeleton of GIF-0726-r and a polyphenol the skeleton of curcumin. The results of in vitro study showed that GIF2165X-G1 (10 μM) increased cell viability, compared to alone oxindole derivative GIF-0726-r or curcumin. Conversely, GIF-2165X-G1 showed lower antioxidant effects compared to curcumin. However, GIF-2165X-G1 has been seen to increase the transcriptional activity of ARE, consequently, the production of antioxidant enzymes such as heme oxygenase-1 (HO-1) is enhanced. In order to confirm the neuroprotective effects demonstrated in vitro study, further investigations were performed on rats injured unilaterally with 6-OHDA in the striatum. GIF-2165X-G1 (1.5 μg) was administered in PD mice. The results showed that these compounds, besides their antioxidant activity, increased TH and DAT levels. Therefore, compound GIF-2165X-G1 shows promise in preserving the functionality of dopaminergic neurons and reducing ROS levels [83].

Instead, M. D. Pandareesh et al. evaluated the neuroprotective effects of curcumin monoglucoside (CMG), bioconjugated curcumin, against ROT-induced toxicity in N27 dopaminergic neuronal cells and *Drosophila* models. Pre-treatment of cells with CMG exerted antioxidant effects by increasing cell GSH levels and decreased ROS. Furthermore, quantitative PCR analysis demonstrated that GMC reduces the upregulation of nitric oxide synthase 2 (NOS2) genes and induced upregulation of NAD(P)H: quinone oxidoreductase 1 (NQO1). Moreover, pretreatment with the synthetic conjugate of curcumin enhanced the activity of the mitochondrial complex I and IV inhibited by ROT. CMG also demonstrated anti-apoptotic effects mediated by a decrease in phosphorylation of JNK3 and c-jun, which induce a reduction of pro-caspase 3. Also in vivo, in the *Drosophila* ROT model, CMG enhanced intracellular antioxidant activity, decreased ROS levels, and prevented dopamine depletion [84]. In line with these results, the antioxidant effects of curcumin have also been demonstrated by Dharmendra K. Khatri et al. In this study, in mice treated with ROT after 21 days of treatment, curcumin reduced MDA and nitrite levels. All three doses of curcumin (50, 100 and 200 mg/kg) used also resulted in a significant reduction in acetylcholinesterase (AChE) activity compared with ROT-treated mice. Conversely, after 21 days, treatment with curcumin at all doses increased the activity of antioxidant enzymes such as SOD and GSH. Furthermore, it reduced oxidative stress also by restoring the activity of the mitochondrial enzyme complex compromised by the ROT. In this way, curcumin improved cognitive function, thus demonstrating its neuroprotective role against PD [85].

Cui Q. et al. demonstrated that pre-treatment with curcumin improved rotational behavior in ROT-treated rats. As shown by Western blot analysis, pretreatment with curcumin decreased ROT-induced loss of TH protein and reduced ROS and MDA production in the substantia nigra pars compacta. Conversely, pre-treatment with curcumin-induced an increase of GSH levels reduced by ROT. Curcumin was shown to exert its antioxidant effects by activating the Akt/Nrf2 signaling pathway in dopaminergic neurons. Indeed, curcumin induced an increase in the expression of the HO-1 and NQO1 proteins and promoted Akt/Nrf2 phosphorylation. In order to confirm this result, rats were infused with an shNrf2 lentivirus or phosphoinositide 3-kinase inhibitor LY294002 prior to treatment with ROT or curcumin. Treatment with shNrf2 or LY294002 inhibited the effects of curcumin, demonstrating that the protective role of curcumin was associated with the Akt/Nrf2 signaling pathway [86].

Nguyen et al. conducted an in vivo experiment using *Drosophila*, which presents ubiquitin carboxyl-terminal hydrolase (dUCH), which is homologous to the human enzyme *UCH-L1* and is useful for reproducing PD. Therefore, targeted studies were carried out on dUCH knockdown flies, in which the oxidative stress condition was highlighted. The authors evaluated the antioxidant properties of curcumin (1 mM). The study was performed both in the larval stage and adult stage; the flies revealed a decrease in ROS in the adult brain and also in the imaginal discs of the eyes. Of considerable interest is that the results found that curcumin improved the motor deficits caused by the knockdown of dUCH. Curcumin has been shown to be relevant in preserving the structure and functionality of dopaminergic neurons by increasing dopamine levels. Notably, a 13% decrease in dopamine was found in curcumin-treated dUCH knockdown flies compared to the control. Instead, 52% of dopamine loss was found in untreated curcumin dUCH knockdown flies compared to the control. Therefore, curcumin confirms its beneficial effects on dopaminergic neurons [87].

The results of these in vivo and in vitro studies have demonstrated that curcuminoids can exert neuroprotective effects both in PD models induced by several environmental factors (such as 6-OHDA, MPTP and ROT), and in PD models induced by genetic factors including α-syn and UCH-L1 (Table 1). Preclinical studies demonstrated that pre-treatment with curcuminoids attenuate the level of oxidative stress and mitochondrial dysfunction. Additionally, curcuminoids prevent α-syn aggregation and fibrillation, consequently, improving the impaired cognitive and motor functions symptoms. To date, there are no available clinical studies that supported the pre-clinical data. Therefore, clinical studies would be necessary to evaluate the efficacy of curcuminoids in patients with PD.

## 5. Alzheimer’s Disease

AD is a neurodegenerative disease that occurs with aging and the cause can be determined by several and concomitant risk factors [88]. Most AD cases occur sporadically. Alone in 10% of cases, AD is caused by genetic mutations in genes such as amyloid precursor protein (*APP*), presenilin 1 (*PSEN1*), and presenilin 2 (*PSEN2*) [89]. AD is characterized by the deposition of extracellular deposits of amyloid-β (Aβ) peptides and neurofibrillary tangles (NFTs) in brain tissue. Under physiological conditions, APP follows the non-amyloidogenic pathway, and is cleaved by α- and γ-secretases [90]. Conversely, in pathological conditions, APP follows the amyloidogenic pathway, which involves the cleavage by two enzymes, β- and γ-secretase, involved in the formation of Aβ. Specifically, Aβ_1–40_ is the most abundant peptide in the brain. In patients with AD, Aβ_1–42_ represents the most abundant and most toxic form due to its predisposition to aggregate and form oligomers [91]. Instead, NFTs are composed of hyperphosphorylated Tau protein, which destabilizes the microtubules inducing tangles formation. The deposition of amyloid plaques and NFTs are the events responsible for the loss of neurons and synapses with consequent impairment of neurotransmission [92,93]. Several studies demonstrate the relationship between oxidative stress and AD development. Indeed, an oxidative imbalance, especially at the mitochondrial level, can cause an increase in ROS, which in turn implies neuronal damage [88,94]. Moreover, it was shown the activation of the N-methyl-D-aspartate receptor (NMDAR) to be involved in oxidative stress, and in particular, in the development and progression of AD. NMDA activation induces an increase in intracellular Ca^2+^, which leads mitochondrial dysfunctions with a consequent increase of oxidative stress [95].

For these reasons, antioxidant therapy in AD patients could be useful. In this context, several studies have been performed on natural compounds such as curcumin, whose antioxidant properties are widely known [96]. Curcumin appears able to inhibit Aβ formation, attenuates the hyperphosphorylation of tau, thus reducing the progression of neuronal damage [97].

### 5.1. Antioxidant Effects of Curcumin in In Vitro AD Model

The efficacy of curcumin was evaluated by Qian et al., in an in vitro experiment on PC12 cells treated with Aβ_25–35_ to reproduce the AD model. The PC12 cells Aβ_25–35_ treated with different doses of curcumin (5, 10, 20, 30 μM/L) showed an increase of cell viability in a dose-dependent manner. Furthermore, the 24 h of pre-treatment with curcumin in a dose-dependent manner has allowed a considerable reduction of apoptosis, through a reduction of caspase 3 expression and increase of Akt phosphorylation. In order to evaluate the antioxidant effects of curcumin, lactate dehydrogenase (LDH) and MDA levels were measured. The curcumin treatment, in a dose-dependent manner, has reduced LDH and MDA levels. Finally, the authors also evaluated that increasing doses of curcumin promoted the expression of NR2A, a subunit of NMDAR, which is important for the functionality of neuronal cells. The authors in this study highlighted the potential neuroprotective and antioxidant role of curcumin in AD [98].

In compliance with these results, Jaroonwitchawan et al. evaluated the ability of curcumin to reduce Aβ production and oxidative stress in SH-SY5Y cells exposed to paraquat. SH-SY5Y cells were treated with paraquat (0.5 mM) for 24 h, evaluating the effects on the expression of genes involved in AD progression. In detail, the paraquat treatment showed an increase in the mRNA transcription of *APP* and *PSEN1* genes. Following 2 h of pre-treatment with curcumin (5 and 10 μM), it was observed a significant reduction of *APP* expression and APP proteins. Additionally, a significant reduction in the Bax/Bcl-2 ratio induced by curcumin would evidence the antiapoptotic effects of this compound. Furthermore, the pre-treatment with curcumin highlighted the antioxidant potential through an increase of SOD and GSH-Px levels. Noteworthy, curcumin enhanced autophagy activity by upregulating LC3I/II. As it is known that the impairment of the autophagic process could play a key role in the processing of APP, the improvement of this process could be a mechanism of action used by curcumin to protect against neurodegeneration [99].

Also, the neuroprotective effects of curcumin were evaluated by Shi et al. in a study conducted on mouse hippocampal HT-22 neuronal cells treated with acrolein to reproduce the AD model. The pre-treatment with curcumin (5 μg/mL) for 30 min showed an increase in cell viability and a decrease in the apoptotic process, reducing the neurotoxic effects of acrolein. Furthermore, pre-treatment with curcumin induced an increase in SOD, GSH levels and showed a reduction in MDA levels in acrolein-treated HT-22 cells. On the other hand, curcumin is able to counteract BDNF/TrkB signaling inhibition induced by acrolein toxicity. The results confirmed curcumin as a neuroprotective agent against AD. In particular, an increase in α-secretase (ADAM-10) was observed, which facilitates the breakdown of APP. Simultaneously, beta-amyloid converting enzyme 1 (BACE1) increased by acrolein exposure was restored [100].

Morales et al. carried out a study to evaluate the antioxidant properties of curcumin in N2a neuroblastoma cells after exposure to cytotoxic compounds. N2a cells were treated separately with ferric nitrilotriacetate and H_2_O_2_, and subsequently treated with curcumin (5–15 μM). Curcumin treatment showed an increase in cell viability, thus confirming cytoprotective effect in neuronal cells. In addition, the researchers incubated human tau (htau40) in N2a cells to reproduce the AD model. N2a cells were incubated for 8 days with monomeric htau40 and heparin to form Tau aggregates. The experimental results were visualized by means of thioflavin-S fluorescence analysis, directly proportional to the aggregated Tau concentration. Simultaneous treatment of curcumin and heparin after one day of incubation showed a reduction in fluorescence, thus a reduction in Tau aggregates. However, the administration of curcumin three days after heparin treatment alone showed a drastic reduction in fluorescence, demonstrating an incisive action against already formed Tau aggregates [101].

In consideration of these findings, Buccarello et al. evaluated the antioxidants properties of curcumin in H_2_O_2_-treated SH-SY5Y cells. The cells were pre-treatment with curcumin (1, 2.5, 5, 10 and 15 μM) for 24, followed by treatment with 0.5 mM of H_2_O_2_ for 30 min. The LDH dosage and cell viability showed that curcumin protected cells against oxidative stress induced by H_2_O_2_. Pre-treatment with curcumin-induced a decrease of caspase-3 level (at the high doses) and LC3B II/I ratio (at each dose tested). Conversely, cells treated with 10 μM of curcumin showed increased ubiquitin levels. Moreover, it was observed that curcumin caused a significant decrease of SUMO-1ylation, the phosphorylation of c-JNK and ERK. Additionally, among the doses of curcumin, only 5 μM induced a significant decrease of Tau phosphorylation compared to control, demonstrating the role of curcumin in the prevention of Tau phosphorylation. Interestingly, immunofluorescence analysis showed that pre-treatment with curcumin (5 μM) reduced the co-localization of SUMO-1-p-JNK-Tau proteins in nuclear bodies induced by H_2_O_2_ treatment [102].

The efficacy of curcumin treatment against oxidative stress was evaluated in macrophages of patients with AD. Jairani et al. conducted an experiment in human monocytic THP-1 cells derived from acute monocytic leukemia, subsequently differentiated into macrophages. Macrophages were treated with H_2_O_2_ (500 µM) to reproduce the AD model. The findings showed less efficient phagocytosis in H_2_O_2_ treated macrophages. Subsequently, macrophages were incubated overnight with HiLyte Flour 488-labeled Aβ_1–42_ (1 µg/mL) to evaluate Aβ_1–42_ internalization. Additionally, the lysosomal marker was used to evaluate Aβ_1–42_ internalization into lysosomes. Therefore, the authors treated the macrophages with curcumin (10 µM). Curcumin improved Aβ_1–42_ internalization in macrophages and lysosomal localization. The investigation also evaluated the presence of Apolipoprotein E (APOE) polymorphisms in AD patients. APOEε3 patient macrophages treated with curcumin internalize more Aβ_1–42_ than APOEε4 patients. Therefore, curcumin ameliorated phagocytic activity in macrophages by preventing neurodegeneration [103].

A recent study illustrated the protective effect of curcumin in the SH-SY5Y cells transfected with the *APPswe* gene, a Swedish mutation, which causes an accumulation of Aβ peptides. The cells were treated with curcumin (0.625–5 μM) for 4 h and subsequently exposed with H_2_O_2_ (250 μM) for 24 h to induce oxidative stress. Curcumin treatment enhanced cell proliferation and reduced LDH release, showing that it decreased H_2_O_2_-induced cell damage. Curcumin reduced the structural changes of neuronal cells, causing less condensation of chromatin with a consequent reduction in the apoptotic process. In order to evaluate the damage induced from oxidative stress to mitochondrial function, it was observed that curcumin was able to decrease the damaging activity of the electron transport chain and reduced the H_2_O_2_–induced mitochondrial membrane depolarization. Oxidative stress has been shown to influence the expression of the *APP* and *BACE1* genes which, conversely, was restored by curcumin. Moreover, curcumin prevented the APP β-cleavage, and intracellular Aβ generation stimulated from H_2_O_2_. Therefore, the antioxidant effects of curcumin, also able to reduce intracellular Aβ, strengthen the hypothesis that it can be used to treat AD [104].

In compliance with the previous study, Yan et al. also showed that curcumin (6.25–25 µM) reduced H_2_O_2_-induced oxidative stress in neuronal PC12 cells. However, curcumin, in addition to reducing ROS levels, is also capable of chelating several metal ions. Indeed, it has been shown that curcumin complexes with metallic ions work in a similar way to SOD. In this context, the authors investigated the protective effects of curcumin-Cu^2+^ or -Zn^2+^ complexes against injury in PC12 cells induced from H_2_O_2_. It was found that the curcumin-Cu^2+^ complex increased cell viability compared to the curcumin or curcumin-Zn^2+^ complex. Furthermore, the curcumin-Cu^2+^ complex showed a rapid increase in the levels of antioxidant enzymes such as SOD, CAT, GSH-Px and it decreased the level of MDA, caspase-3, and caspase-9. On the other hand, curcumin and curcumin-Cu^2+^ or -Zn^2+^ complexes increased the Bcl-2/Bax ratio, and reduced the level of NF-κB p65, demonstrating that curcumin suppresses apoptosis. Therefore, these results highlight the potential therapeutic value of curcumin complexed with metal ions in AD [105].

However, curcumin is known to have low bioavailability, which makes it difficult to clearly understand its pharmacological effects. Djiokeng Paka et al., in order to increase the bioavailability of this compound, carried out an in vitro experiment through the encapsulation of curcumin inside poly (lactide-co-glycolide) (PLGA) nanoparticles (NPs) with a ratio of 50% lactic acid (LA) and 50% glycolic acid (GA) (NPs-Curcumin 50:50) or with a ratio of 65% LA and 35% GA (NPs-Curcumin 65:35). SKN-SH cells were treated with free curcumin (0.5 µM), NPs-Curcumin 50:50 and NPs-Curcumin 65:35 for 1 h. The findings showed good absorption of NPs-Curcumin 50:50 in neuronal cells. In order to evaluate the antioxidant effects of curcumin, the cells were exposed to H_2_O_2_. NPs-Curcumin 50:50 significantly reduced ROS levels. Therefore, the authors focused their attention on the Nrf2/Keap1 pathway showing that the treatment with free curcumin (0.5 µM) and NPs-Curcumin 50:50 in SK-N-SH H_2_O_2_-treated cells reduced the activation of Keap1 and consequently of Nrf2 activation. 

Oxidative stress plays a key role also in Akt and Tau phosphorylation. In this case, NPs-Curcumin 50:50 have been shown to be effective in reducing their phosphorylation. It was also evaluated the change expression of genes that play an important role in antioxidant and neuroprotective processes. In particular, NPs-Curcumin increased transcripts of glutaredoxine (*GLRX*), thioredoxine (*TRX*), and a decrease in apolipoprotein J (*APOJ*). Both NPs-Curcumin 50:50 and NPs-Curcumin 65:35 appear more effective than free curcumin in modulating these genes. In conclusion, the use of curcumin encapsulated in PLGA nanoparticles could be a valid therapeutic strategy to overcome the problems of the clinical application of curcumin related to its poor bioavailability [106].

The poor stability and low bioavailability of curcumin are due to β-diketone moiety that induces rapid degradation. In this context, two mono-carbonyl analogues of curcumin, (1E, 4E)-1,5-bis(4-hydroxy-3-methoxyphenyl)penta-1,4-dien-3-one (CB) and (1E, 4E)-1-(3,4-dimethoxyphenyl)-5-(4-hydroxy-3, 5-dimethoxyphenyl) Penta-1, 4-dien-3-one (FE) were synthesized. PC12 cells were treated with Aβ_25–35_ (10 μM) before, concurrently, or after treatment with curcumin, CB, and FE at different concentrations (0.1–20 μM). Treatment with CB and FE showed improvements in cell viability and counteracted the increase in ROS following Aβ_25–35_ induced toxicity. Additionally, CB and FE have been shown to be effective in restoring levels of antioxidant enzymes such as CAT and SOD. Significant reductions in MDA and LDH dosages were also found following treatment with curcumin and analogues. CB and FE also resulted in an increase in the Bcl2/BAX ratio and a reduction in cytochrome c release as a result of inhibition of apoptosis. However, proteins of the Keap1/Nrf2/HO-1 signaling pathway were evaluated in PC12 cells, a key pathway to protect the cells from oxidative stress and apoptosis. Curcumin, CB, and FE reduced Keap1 expression and simultaneously increased Nrf2 and HO-1 expression. This study highlighted that the mono-carbonyl analogues of curcumin showed great efficacy at lower doses compared to curcumin. This demonstrated that the CB and FE used a similar mechanism of curcumin, and they showed major stability. Thus, the modification of mono-ketone moiety could improve the stability bioavailability of curcumin. In conclusion, the results of this study demonstrated mono-carbonyl analogues of curcumin might be implicated in the treatment of AD [107].

Instead, Pinkaew et al. evaluated the neuroprotective effects di-O-demethylcurcumin, a modified analog of curcumin. In this study, SK-N-SH cells were pre-treated with di-O-demethylcurcumin (1–8 μM) for 2 h and then incubated with Aβ_25–35_ (10 μM) over-night. The di-O-demethylcurcumin pre-treatment showed a reduction in cell toxicity and in ROS and NO levels compared to Aβ_25–35_ group. The pretreatment with di-O-demethylcurcumin downregulated iNOS expression, thereby reducing NO production. Further, di-O-demethylcurcumin exposure increased Nrf2 protein expression in the nucleus with a consequent increase of pathway-related proteins such as HO-1, NQO1, and SOD. Additionally, di-O-demethylcurcumin showed anti-inflammatory properties avoiding the translocation of NF-kB p65 into the nucleus. Therefore, di-O-demethylcurcumin could be a valid candidate against Aβ_25–35_-induced neurotoxicity [108].

The curcumin derivatives behavior was also explored by Orteca et al. in hippocampal HT-22 mouse cells. In order to improve bioavailability and curcumin stability, the researchers modified the molecule of curcumin through the removal of the keto-enol fraction, the addition of pyrazole ring, or insertion of the phthalimide-functionalized chain. To induce neurotoxicity, HT-22 cells were treated with glutamate (2 μM) and subsequently were co-treated with curcumin and curcumin derivatives (1 µM) for 24 h. The curcumin derivatives compared to curcumin showed a greater reduction in the cytotoxic effects and the apoptotic process induced by the treatment with glutamate. Additionally, curcumin derivatives downregulated iNOS and decreased the ratio of Bax/Bcl2 transcripts, thus confirming their cytoprotective and antiapoptotic actions against oxidative stress. Further, fluorescence analysis was done to study the interaction between curcumin derivatives and amyloid fibrils. In this regard, HT-22 cells were treated with Aβ_1–40_ (10 μM) to induce the AD model. The treatment with curcumin derivative (10 μM) for 24 h, displayed that these compounds own higher binding affinity and depolymerization of fibrillar aggregates compared to curcumin. Therefore the curcumin-derived compounds exhibited a high bioavailability compared to curcumin, and they reveal satisfactory ability to counteract oxidative stress and depolymerize fibrillar aggregates [109].

### 5.2. Antioxidant Effects in In Vivo AD Model

The effects of curcumin on behaviors and biochemical markers related to AD-like symptoms were investigated in vivo experimental model AD. The AD model was induced by bilateral hippocampal injection of streptozotocin (3.0 mg/kg), associated with subcutaneous administration of D-galactose (125 mg/kg) for 7 weeks, which served to promote neurodegeneration and increase oxidative stress. Rats were treated with curcumin (10 mg/kg) via intraperitoneal injection for 7 weeks. After treatment, an increase in GSH-Px enzymatic activity was observed in the blood samples of the curcumin-treatment group compared to the AD groups. Curcumin reduced oxidative stress damage induced by the combination of streptozotocin and D-galactose. The histochemical investigations allowed it to visualize the effects of curcumin-mediated treatment in the cortex and hippocampus regions CA1 and CA3. The curcumin-mediated treatment avoided a substantial loss of neurons in the hippocampal tissue. Moreover, curcumin reduced the APP β-cleavage and formation of amyloid-like and reduced the Aβ_1–42_ in the hippocampal compared to the AD group. Also, in the curcumin group, it was observed the reduction of PSEN1 and BACE1 expression. Therefore, curcumin prevented neurodegeneration and preserved the integrity of hippocampal tissue [110].

Although curcumin has excellent therapeutic potential against AD, its poor bioavailability and biodistribution could reduce its effectiveness. In this context, Fidelis et al. evaluated antioxidant and antidepressant effects using curcumin-loaded lipid core capsules (NLC C) in Swiss male mice reproducing AD models. In order to induce the AD model, aggregated acetyl Aβ_25–35_ (3 nmol/3μL) was administered intracerebroventricularly. Curcumin was administrated via intragastrical, in order to overcome its low bioavailability. The mice were pre-treated with NLC C (10 mg/kg) every 48 h for 12 days. The results of the present study showed that NLC C reduced the levels of ROS and regularized the levels of antioxidant enzymes such as SOD and CAT raised by treatment with Aβ_25–35_ in the prefrontal cortex. However, studies carried out on the hippocampus did not reveal significant differences, but NCL C resulted in an increase in the SOD/CAT ratio. Therefore, NLC C proved to be an effective treatment against oxidative stress induced by Aβ_25–35_ in the prefrontal cortex, and also proved effective in attenuating the depressive behavior induced by Aβ_25–35_ infusion [97].

Also, Malvajerd et al. evaluated the effects of NLC C in male Sprague-Dawley rats Aβ_1–42_-treated to induce a model of AD. The treatment of NLC C (4 mg/kg) administered intracerebroventricularly showed an overall reduction in the oxidative stress induced by the Aβ_1–42_ treatment in the hippocampal tissue of rats. Specifically, the results showed a decrease in MDA, ROS levels, and lipid peroxidation in the hippocampal tissue, demonstrating that curcumin counteracts oxidative stress. Moreover, the researchers found an increase of thiols, peculiar for their antioxidant properties. A significant finding, the ADP/ATP ratio was also assessed, it was decreased in NLC C treatment compared to the sham group. The efficacy of NLC C for brain delivery of curcumin was also confirmed by the improvement of spatial memory in rats. This evidence confirmed the antioxidant properties of curcumin and proved that NLC C to be a good vector for reaching optimal concentrations in the brain [111].

### 5.3. Antioxidant Effects of Curcumin in Clinical Trial

Some clinical studies were conducted in order to investigate the potential effects of the efficacy of CBD in the management of AD.

A group of Chinese researchers performed a phase 1/2 trial (NCT00164749), to evaluate the efficacy of curcumin in slowing the progression of AD. In this study, 36 patients (50 years and older) with a progressive decline in cognitive function and memory for at least six months were enrolled. Patients were treated with a placebo or with 1 or 4 g of curcumin per day. Additionally, all patients were treated with 120 mg of ginkgo extract per day. All participants were monitored at baseline, 1, 3, and 6 months after treatment. The primary outcome of the study was to assess the safety of curcumin in order to be able to design a larger study to test curcumin against AD. The secondary objective of the study was to establish the biochemical mechanism used from curcumin and explain which of the two doses was more effective. Therefore, to assess whether curcumin could reduce levels of Aβ and oxidative stress-related proteins, blood samples were analyzed to measure levels of isoprostane, Aβ protein, metals, and cholesterol. Cognitive tests were performed to evaluate the effects of curcumin on cognitive impairments typical of AD. In conclusion, the results of this study will help us understand whether curcumin can be used as a treatment to delay the progression of AD.

The complete phase 2 study (NCT00099710), double-blind, aimed to evaluate the safety and tolerability of two doses of Curcumin C3 Complex ^®^ in patients with mild or moderate AD. The study enrolled 33 patients (50 years and older) randomized to receive one of two doses of oral curcumin (2 or 4 mg), or a placebo, for the first six months of the study. After six months, patients in the placebo group received one of two doses of curcumin, thereby extending the study to another six months. Participants achieved seven visits during the 12-month study. Physical and neurological examinations, routine laboratory tests, and neuropsychological assessments were performed during these visits. Specifically, Alzheimer’s Disease Assessment Scale, cognitive sub-portion (ADAS-Cog), the Neuropsychiatric Inventory (NPI) tool, and Alzheimer’s Disease Cooperative Study Activities of Daily Living (ADCS-ADL) were used to assess the clinical outcomes of the treatment. Blood and cerebrospinal fluid tests were also done to assess how curcumin is absorbed by the body and its possible antioxidant and anti-inflammatory effects. 

Part of the study results was published in the study conducted by Ringman J. M. et al. In this study, the authors reported only efficacy data at 6 months post-treatment and tolerability measures of curcumin up to 12 months. After one year of treatment, curcumin was well tolerated; of the 33 patients enrolled, only three subjects withdrew from the study due to severe gastrointestinal symptoms. However, the study results did not report clinical data on the efficacy of curcumin against AD. Indeed, there were no significant differences between the treatment groups in ADAS-Cog, NPI, and ADCS-ADL scores. There were also no significant changes in plasma and cerebrospinal fluid levels of Aβ_1–40_, Aβ_1–42_, tau, and p-tau. Additionally, curcumin appears to have shown low bioavailability. In conclusion, the poor bioavailability, the lack of clinical data demonstrating the efficacy of curcumin in AD, and the partial publication of the data, do not allow definitive conclusions to be drawn from this study, further observations and long-term clinical studies will be necessary to understand the mechanisms used by curcumin to exert its beneficial effects.

Due to the poor bioavailability of curcumin, which does not allow for positive results in clinical studies, a phase 2 clinical trial (NCT01001637) was designed to determine the efficacy and safety of the highly bioavailable curcumin, solid-lipid curcumin particle (SLCP or Longvida). The study enrolled 26 participants (50 to 80 Years) with moderate or severe AD. The patients were randomly divided into a group that received a placebo and a group treated with oral curcumin formulation at a dose of 2000 mg or 3000 mg daily. The primary outcome of the study was to evaluate the efficacy of curcumin formulation on the mental abilities of AD patients 2 months after treatment. The trial also aimed to evaluate changes in serum Aβ concentrations. The results of the study have not yet been published, however, they will help to understand the oral dosing of curcumin solid-lipid particles, and to evaluate its safety. Furthermore, data will be helpful to understand whether formulated curcumin induces improvements in memory deficits and influences biomarkers of AD better than non-formulated curcumin.

In conclusion, preclinical findings demonstrated that curcuminoids during pre-treatment inhibit the Aβ aggregation, attenuate the tau hyperphosphorylation and, consequently, improve cognitive function and prevent the development of dementia (Table 2). However, clinical studies that reveal the efficacy of curcuminoids treatment in AD patients with mild or moderate stages of the disease are few. Thus, further studies will be needed to encourage the use of curcuminoids as valid therapeutic tools in the management of AD.

## 6. Amyotrophic Lateral Sclerosis

ALS is a neurodegenerative disease characterized by progressive degeneration of both upper and lower motor neurons, resulting in muscle atrophy, gradual paralysis that generally results in respiratory failure [112]. Respiratory failure usually occurs within 2–5 years of diagnosis due to respiratory muscle involvement. Furthermore, about 50% of ALS patients show evidence of frontal and temporal lobe dysfunction, while about 15% of patients with ALS, show some degree of motor involvement [113]. About 10% of patients with ALS follow a family pattern, mostly autosomal dominant (familial ALS), while 90% show a genetic basis (sporadic ALS). Several gene mutations linked to ALS have been associated to date, including superoxide dismutase 1 (SOD1), TAR DNA-binding protein 43 (TDP43), and fused in sarcoma/translocated in sarcoma (FUS) genes. The etiology of ALS still remains completely misunderstood, however like other neurodegenerative diseases it is a multifactorial pathology [114]. Certainly, glutamate-induced excitotoxicity, microglia activation, apoptosis, neuroinflammation, oxidative stress and mitochondrial dysfunction are all factors that play a key role in the pathogenesis of this disease [115,116].

Several studies have demonstrated the important role that oxidative stress plays a key role in the development and progression of ALS. Indeed, oxidative damage due to ROS accumulation has been confirmed in NSC-34 cells expressing mutant or wild-type TDP-43 genes [117]. Oxidative stress is responsible for alterations in redox signaling that exacerbate other pathophysiological processes such as excitotoxicity [118], mitochondrial damage [119], alterations in astrocyte and microglia signaling [120,121], responsible for damage to motor neurons. Treatment with antioxidants compounds may slow the progression of the disease in animal models of ALS [122,123]. Curcumin, in motor neuron cell lines transfected with the TDP-43 mutant, reduces oxidative stress and protects cells from mitochondrial damage [124,125]. Furthermore, curcumin activates Nrf2 target genes in primary astrocytes of the spinal cord. Since Nrf2 is a regulator of antioxidant genes, the activation of this antioxidant pathway may be a valid therapeutic strategy used by curcumin to prevent the loss of motor neurons in the spinal cord [126].

In this regard, Chico L. et al. evaluated the effects of curcumin in patients with ALS. Patients were randomized into two groups, one group received a placebo for three months and subsequently received oral curcumin (600 mg/day, Brainoil), for three months. While a second group was treated with curcumin for six months (600 mg/day, Brainoil). Evaluations were performed at baseline after three months and after six months. Clinical evaluations were performed and biomarkers of oxidative stress were measured, including oxidative protein products (AOPP), ferric reducing capacity (FRAP), total thiols (T-SH), and lactate which were compared with a control group. Throughout the entire study, the group that received curcumin from the outset showed a stable Revised Amyotrophic Lateral Sclerosis Functional Rating Scale (ALSFRS-R) score. Furthermore, this group always showed a reduction in AOPP (*p* < 0.01), which was not observed in group A. Curcumin administered for six months also maintained stable FRAP and decreased lactate. Furthermore, the patients with ALS recruited in the study showed an increase in oxidative stress compared to healthy subjects. However, treatment with curcumin reduced oxidative stress. Thus oral supplementation of curcumin revealed encouraging data indicating a slight slowdown in disease progression, ameliorating aerobic metabolism, and reducing oxidative damage [127].

The effects of curcumin are also being evaluated in another clinical trial still under study. The ongoing trial registered in ClinicalTrials.gov (accessed on 12 February 2021) (NCT04654689), intends to evaluate the effects of curcumin used in combination with another polyphenol, resveratrol. The study was recruited 100 ALS patients (over 18 years of age) for at least 6 months that were randomized into two groups. One group will receive a single daily dose of the combination of resveratrol (75 mg) and curcumin (200 mg) liposomed with dutasteride for 6 months. The second group will receive placebo at the same dose and for the same time frame. The placebo will consist of sucrose as a replacement for liposomal polyphenols, and a soft capsule of microcrystalline methylcellulose, instead of Dutasteride. During the study, assessments will be performed at baseline, three months, and six months after treatment. Patients will undergo clinical evaluations using functional, cognitive, and behavioral tests through ALSFRS-R, electromyography, and measurement of forced vital capacity evaluations. 

Furthermore, to evaluate the possible antioxidant and anti-inflammatory effects induced by the treatment, quantitative plasma measurements of inflammatory cytokines such as plasma IL-6 and TNF-α and of oxidative stress markers such as Trolox Equivalent Antioxidant Capacity (TEAC), 8-oxoguanine and MDA. The choice of using these compounds in liposomes or nanoparticles increases their stability, bioavailability, and absorption of the antioxidant. Indeed, the use of nanobiotechnology with curcumin (80 mg/day) in the treatment of patients with ALS has obtained positive outcomes demonstrating that nanocurcumin is safe and could improve the course of the disease, as well as proving to be a valid therapeutic strategy to use as an adjunct treatment in patients with ALS [128]. Therefore, the results of this clinical study will be useful in understanding the mechanisms by which these compounds exert their protective effects. Additionally, they will also be necessary to assess if natural compounds such as curcumin could be a valid therapeutic strategy to use in patients with ALS.

These results suggest that curcuminoids, through their antioxidant effects, are able to prevent the loss of motor neurons. Interestingly, the treatment of ALS patients with curcumin is safe and useful in improving and preventing the progression of the disease. Therefore, these data encourage the use of curcuminoids as a therapeutic strategy in the treatment of ALS.

## 7. Conclusions

**Figure 3:**
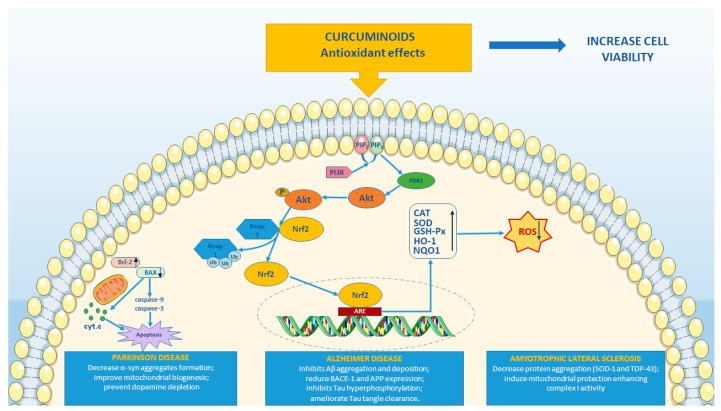
Graphical representation illustrates the principal antioxidant effects of curcuminoids in Parkinson’s disease (PD), Alzheimer’s disease (AD) and amyotrophic lateral sclerosis (ALS). The figure was made taking the images from Servier Medical Art (available at http://smart.servier.com/ accessed on 12 February 2021), licensed under a Creative Commons Attribution 3.0 Unported License (https://creativecommons.org/licenses/by/3.0/ accessed on 12 February 2021). PIP_2_, Phosphatidylinositol 4,5-bisphosphate; PIP_3_, Phosphatidylinositol (3,4,5)-trisphosphate; PDK1, Phosphoinositide-dependent kinase-1; PI3K, Phosphoinositide 3-kinase; Akt, Protein kinase B; Keap1, Kelch-like ECH-associated protein 1; Ub, Ubiquitin; P, phosphate; Nrf2, Nuclear factor erythroid 2-related factor 2; ARE Antioxidant response element; CAT, catalase; SOD, superoxide dismutase; GSH-Px, glutathione peroxidase; HO-1, heme oxygenase-1; NQO1, NAD(P)H quinone oxidoreductase 1; ROS, Reactive oxygen species; Bcl-2, B-cell lymphoma 2; BAX, bcl-2-like protein 4; cyt. c, cytochrome complex; α-syn, alpha-synuclein; Aβ amyloid-β; BACE1, beta-amyloid converting enzyme 1; Tau, Tau protein; APP, Amyloid precursor protein; SOD1, superoxide dismutase 1; TDP43, TAR DNA-binding protein 43.

This review summarized the studies that evaluate the antioxidant properties of curcuminoids related to their potential neuroprotective effects. Specifically, curcuminoids highlight powerful antioxidant actions through the activation of Akt/Nrf2 pathway (Figure 3). Several preclinical studies have shown that curcuminoids possess therapeutic efficacy against AD, PD and ALS. However, there are not available clinical studies for PD. Instead, few clinical trials are recorded concerning AD and ALS that limited results reported efficacy and safety of curcuminoids. Therefore, clinical studies are needed in order to include curcuminoids in clinical practice.

## Figures and Tables

**Table 1:** Antioxidant effects of Curcuminoids in PD.

Models	Curcuminoids	Curcuminoids Dose	Treatments	Mechanism of Action	Ref.
			In vitro experimental models		
MPP^+^ Stimulated primary mesencephalic astrocytes	Curcumin	8 μM	28 h of treatment	Pre-treatment with curcumin reduced the oxidative stress in primary mesencephalic astrocytes induced by MPP^+^ -treatment. Therefore, curcumin decreased ROS and increased GSH compared to primary astrocytes treated with MPP^+^.	[77]
Model of oxidative stress in SH-SY5Y neuronal cells induced by the H_2_O_2_	Curcumin	5 µM	24 h of treatment	Curcumin reduced lipid peroxidation and intracellular Ca^2+^ concentrations; conversely enhanced GSH and GSH-Px levels compared to the H_2_O_2_-treated group of cells. In cells treated with curcumin caspase-3 and caspase-9 expression were reduced.	[78]
Mouse brain mitochondria treated with α-syn and hen egg white lysozyme	Curcumin	25 and 50 μM	Mitochondrial homogenates were pre-incubated with curcumin for 30 min at 30 °C, prior to adding α-syn fibrillation products	Curcumin, in a dose-dependent manner, reduced the growth of α-syn fibrils in rat brain mitochondria, by preventing mitochondrial Type 1 Hexokinase release and ROS formation.	[79]
SH-SY5Y ROT-treated	DMC	50 nM	2 h of pre-treatment	DMC inhibited the apoptosis process reducing pro-apoptotic proteins, increasing anti-apoptotic proteins, and ameliorating oxidative stress.	[80]
PC12 ROT-treated	Curcumin	10 μM	24 h of co-treatment	Curcumin increased cell viability and reduced oxidative stress. Treatment also decreased carbonylation and protein tyrosine nitration. Curcumin, prevent proteasome degradative system ROT-induced.	[81]
			In vivo experimental models		
Adult male Sprague-Dawley rats injured unilaterally with 6-OHDA in the left striatum	Curcumin	10 µmol/L	Intragastrically administration once each day for 3 consecutive weeks	Curcumin induced a significant increase in Wnt3a and β-catenin mRNA expression. It increased levels of GSH-Px, SOD, and reduced MDA. Curcumin reduced the local tissue damage induced by 6-OHDA, increasing the levels of the DAT, TH, and reducing GFAP.	[82]
HT-22 cells glutamate-induced and adult C57Bl/6 mice injuried unilaterally with 6-OHDA in the right striatum	GIF-2165X-G1	10 μM (in vitro); 1.5 μg (in vivo)	HT-22 cells pre-treated with GIF-2165X-G1 for 24 h; mice were co-treated with injections of GIF-2165X-G1 and 6-OHDA in the right striatum	GIF-2165X-G1 in HT-22 neuronal cells increases cell viability. GIF-2165X-G1 increased the transcriptional activity of ARE, consequently, enhanced antioxidant enzymes such as HO-1. Instead, in mice, besides its antioxidant activity, GIF-2165X-G1 increased TH and DAT levels, improving the functionality of dopamine neurons.	[83]
N27 cell and *Drosophila* ROT-induced PD models	CMG	0.25−5 µM (in vitro); 500 µM (in vivo)	N27 cells pre-treated with CMG for 24 h; flies were pre-treated with CMG via diet for 5 days	CMG increased glutathione levels and decreased ROS. CMG upregulated the expression of NOS2 and downregulated the NQO1. CMG reduced the phosphorylation of JNK3 and c-jun, with a consequent decrease of pro-caspase 3. The same results were also obtained in the *Drosophila* ROT model.	[84]
ROT-treated Swiss albino male mice	Curcumin	50, 100 and 200 mg/kg	Orally administration for 21 days 1 h before ROT treatment	Curcumin (50, 100 and 200 mg/kg) increased SOD and GSH; instead, it reduced MDA, nitrite levels and the AchE activity. Curcumin improved the activity of the mitochondrial enzyme complex and ameliorated cognitive function.	[85]
ROT-treated male Lewis rats	Curcumin	100 mg/kg	Intragastrically administration twice a day for 50 days, before to ROT administration	Pretreatment with curcumin decreased ROT-induced loss of TH protein, ROS and MDA production and increased GSH levels through activation of the Akt/Nrf2 signaling pathway in dopaminergic neurons.	[86]
*Drosophila* model with dUCH Knockdown	Curcumin	1 mM	-	Curcumin lowered ROS levels in the brain and imaginal discs of the eyes. It also has beneficial effects on dopaminergic neurons, preserving their structure and functionality	[87]

MPP^+^, 1-methyl-4-phenylpyridinium ion; ROS, reactive oxygen species; GSH, glutathione; H_2_O_2_, hydrogen peroxide; GSH-Px, glutathione peroxidase; α-syn, α-synuclein; ROT, rotenone; PD, Parkinson’s disease; DMC, demethoxycurcumin; 6-OHDA, 6-hydroxydopamine; SOD, superoxide dismutase; MDA, malondialdehyde; DAT, dopamine transporter; TH, tyrosine hydroxylase; GFAP, glial fibrillar acid protein; CMG, curcumin monoglucoside; NOS2, nitric oxide synthase 2; NQO1, NAD(P)H: quinone oxidoreductase 1; JNK3, c-Jun N-terminal kinase; AChE, acetylcholinesterase; dUCH, ubiquitin carboxyl-terminal hydrolase.

**Table 2:** Antioxidant effects of curcuminoids in AD.

Models	Curcuminoids	Curcuminoids Dose	Treatments	Mechanism of Action	Ref.
In vitro experimental models					
PC12 cells treated with Aβ_25–35_	Curcumin	5, 10, 20, 30 μM/L	24 h of pre-treatment	Curcumin increased the cell viability in a dose-dependent manner and reduced apoptosis, through caspase 3 expression reduction and Akt phosphorylation increase. The treatment, in a dose-dependent manner, has reduced LDH and MDA levels. Conversely, treatment enhanced the expression of NR2A, a subunit of NMDAR, highlighting the antioxidant properties of curcumin in AD.	[98]
SH-SY5Y paraquat-treated	Curcumin	5 and 10 μM	2 h of pre-treatment	Curcumin reduced APP expression and APP proteins. This compound also reduced the ratio Bax/Bcl-2 induce by curcumin, evidencing the antiapoptotic effects. While the antioxidants effects were highlighted from increased SOD and GSH-Px levels. Curcumin enhanced autophagy activity by upregulating LC3I/II.	[99]
HT-22 treated with acrolein	Curcumin	5 μg/mL	0.5 h of pre-treatment	Curcumin induced an increase in the levels of SOD, GSH and a reduction in the levels of MDA. Furthermore, curcumin is able to counteract BDNF/TrkB pathway inhibition.	[100]
N2a cells treated with ferric nitrilotriacetate or H_2_O_2_ or htau40	Curcumin	5–15 μM	24 h or 1 and 3 days after htau40 treatment.	Curcumin showed increased cell viability induced by ferric nitrilotriacetate or H_2_O_2_. Furthermore, the curcumin treatment reduced Tau aggregates.	[101]
SH-SY5Y cells treated with H_2_O_2_	Curcumin	1, 2.5, 5, 10 and 15 μM	24 h of pre-treatment	Curcumin increased cell viability after H_2_O_2_ treatment. in addition, it decreased caspase 3 levels and the LC3B II/I ratio. Moreover, curcumin (5 μM) reduces Tau phosphorylation and co-localization of SUMO-1-p-JNK-Tau proteins in nuclear bodies, demonstrating neuroprotective effects.	[102]
Macrophages treated with H_2_O_2_ or Aβ_1–42_	Curcumin	10 µM	24 h of treatment	Curcumin enhanced the phagocytic activity of macrophages through the internalization of Aβ _1–42_ into lysosomes. Therefore, curcumin prevents neurodegeneration.	[103]
SH-SY5Y cells transfected with the APPswe gene and exposed to H_2_O_2_	Curcumin	0.625–5 μM	4 h of pre-treatment	Curcumin ameliorated cell proliferation and decreased LDH release. Treatment reduced the structural changes, preventing the apoptotic process. Curcumin inhibited oxidative stress-induced damage to impaired mitochondrial function. It restored the expression of the APP and BACE1 genes and avoided the APP β-cleavage and intracellular Aβ generation.	[104]
Model of oxidative stress in PC12 cells induced by the H_2_O_2_	Curcumin, curcumin-Cu^2+^ complexes and curcumin-Zn^2+^ complexes	0.5 µM	0.5 h of pre-treatment	Curcumin-Cu^2+^ or -Zn^2+^ complexes enhanced the levels of antioxidant enzymes such as SOD, CAT, GSH-Px and decreased the MDA levels. Both complexes suppressed apoptosis through reduced caspase-3, caspase-9, and NF-κB p65 levels; conversely, it increased the Bcl-2/Bax ratio. Compared to the curcumin or curcumin-Zn^2+^ complex, curcumin-Cu^2+^ appeared most efficacy also in the increase of cell viability.	[105]
Model of oxidative stress in SK-N-SH cells induced by the H_2_O_2_	NPs-Curcumin formulations	0.5 µM	1 h of treatment	NPs-curcumin 50:50 significantly reduced ROS levels inhibiting the Keap1 and Nrf2 activation and reducing the phosphorylation of Akt and Tau. NPs-curcumin appears more effective than free curcumin in modulating the expression of genes that play an important role in antioxidant and neuroprotective processes, such as *GLRX*, *TRX*, and *APOJ*.	[106]
PC12 Aβ_25–35_-treated	Curcumin, CB And FE	0.1–20 μM	-	Treatment with CB and FE improved cell viability and counteracted the increase in ROS. Both mono-carbonyl analogues of curcumin restored levels of antioxidant enzymes (such as CAT and SOD) and reduced MDA and LDH. CB and FE also increased the Bcl2/BAX ratio and reduced cytochrome c release as a result of inhibition of apoptosis. Curcumin, CB, and FE reduced Keap1 expression and simultaneously increased Nrf2 and HO-1 expression. The mono-carbonyl analogues of curcumin showed great efficacy at lower doses compared to curcumin.	[107]
SK-N-SH Aβ_25–35_-treated	Di-O-demethylcurcumin	1–8 μM	2 h of pre-treatment	The di-O-demethylcurcumin reduced ROS and iNOS expression, thus decreasing NO production. The di-O-demethylcurcumin exposure increased Nrf2 protein expression in the nucleus with a consequent increase of pathway-related proteins such as HO-1, NQO1, and SOD.	[108]
HT-22 cells glutamate-treated or Aβ_1–40_-treated	Curcumin and curcumin derivatives	1 µM and 10 µM	24 h of co-treatment	Curcumin derivatives showed reduced cytotoxic effects and apoptotic processes. Indeed, curcumin derivatives downregulated iNOS and reduced the ratio of Bax/Bcl2 transcripts. Moreover, curcumin derivatives exhibited higher binding affinity and depolymerization of fibrillar aggregates, thus they showed neuroprotective effects.	[109]
In vivo experimental models					
Sprague-Dawley rats streptozotocin and D-galactose co-treated	Curcumin	10 mg/kg	Intraperitoneal injection for 7 weeks	Curcumin increased the GSH-Px reducing oxidative stress damage induced by the combination of STZ and D-galactose. The treatment ameliorated the loss of neurons in the hippocampal tissue. Curcumin reduced the AβPP β-cleavage, formation of amyloid-like, and reduced the Aβ_1–42_ in the hippocampal. It also decreased the PSEN1 and BACE1 expression.	[110]
Swiss male mice-treated Aβ_25–35_	NLC C	10 mg/kg	Intragastrically administration once every 48 h for 12 days	NLC C restored SOD and CAT concentrations in the prefrontal cortex. In the hippocampus it was observed that NCL C increased the SOD/CAT ratio.	[97]
Sprague-Dawley male rats-treated Aβ_1–42_	NLC C	4 mg/kg	Intracerebro-ventricular administration for 4 days after Aβ_1–42_ injection	NLC C reduced ROS and MDA levels and increased the concentration of thiol groups, indicating a contrasting action in oxidative stress. It also preserved mitochondrial functionality. NLC C promoted the restoration of cognitive functions.	[111]

Aβ, amyloid-β; LDH, lactate dehydrogenase; MDA, malondialdehyde; NMDAR, N-methyl-D-aspartate receptor; APP, amyloid precursor protein; BACE1, beta-amyloid converting enzyme 1; SOD, superoxide dismutase; GSH-Px, glutathione peroxidase; H_2_O_2_, hydrogen peroxide; JNK, Jun N-terminal kinase; Cu^2+^, copper; Zn^2+^, zinc; CAT, catalase; NF-κB, nuclear factor κB; NPs, nanoparticles; GLRX, glutaredoxine; TRX, thioredoxine; APOJ, apolipoprotein J; PSEN1, presenilin 1; NLC C, curcumin-loaded lipid core capsules; CB, (1E, 4E) -1, 5-bis (4-hydroxy-3-methoxyphenyl) penta-1, 4-dien-3-one; FE, (1E, 4E)-1-(3,4-dimethoxyphenyl)-5-(4-hydroxy-3,5-dimethoxyphenyl) Penta-1, 4-dien-3-one; HO-1, heme oxygenase-1; NQO1, NAD(P)H quinone oxidoreductase 1.

## Data Availability

No new data were created or analyzed in this study. Data sharing is not applicable to this article.
